# Recent Advances in Processing of Titanium and Titanium Alloys through Metal Injection Molding for Biomedical Applications: 2013–2022

**DOI:** 10.3390/ma16113991

**Published:** 2023-05-26

**Authors:** Al Basir, Norhamidi Muhamad, Abu Bakar Sulong, Nashrah Hani Jamadon, Farhana Mohd Foudzi

**Affiliations:** Department of Mechanical and Manufacturing Engineering, Faculty of Engineering and Built Environment, Universiti Kebangsaan Malaysia, Bangi 43600, Selangor, Malaysia; al.basir005@yahoo.com (A.B.); norhamidi@ukm.edu.my (N.M.); nashrahhani@ukm.edu.my (N.H.J.); farhana.foudzi@ukm.edu.my (F.M.F.)

**Keywords:** titanium and titanium alloys, metal injection molding, biomedical, sintering, mechanical properties

## Abstract

Metal injection molding (MIM) is one of the most widely used manufacturing processes worldwide as it is a cost-effective way of producing a variety of dental and orthopedic implants, surgical instruments, and other important biomedical products. Titanium (Ti) and Ti alloys are popular modern metallic materials that have revamped the biomedical sector as they have superior biocompatibility, excellent corrosion resistance, and high static and fatigue strength. This paper systematically reviews the MIM process parameters that extant studies have used to produce Ti and Ti alloy components between 2013 and 2022 for the medical industry. Moreover, the effect of sintering temperature on the mechanical properties of the MIM-processed sintered components has been reviewed and discussed. It is concluded that by appropriately selecting and implementing the processing parameters at different stages of the MIM process, defect-free Ti and Ti alloy-based biomedical components can be produced. Therefore, this present study could greatly benefit future studies that examine using MIM to develop products for biomedical applications.

## 1. Introduction

The history and development of biomaterials are long and rich. Archaeologists have discovered that biomaterials have been used as dental implants since as early as 200 A.D. [1,2,3]. According to Vallet-Regí [4], after World War II, multiple materials were examined to create prostheses and implants for soldiers wounded in action. At that time, the term “biocompatibility” solely described how well an organism tolerated a material. However, in the 1960s, some of these implants led to complications. This created a need to identify implant materials that did not cause more problems than they effectively solved, as well as how to implant these new materials. In the late 1960s, surgical, medical, and dental laboratories began examining effective methods of producing replacement parts and implants for bodily injuries and published their findings in biomedical literature. Since then, the prevalence of such implants has increased globally.

Biomaterial implants are commonly used to replace and restore damaged or deteriorated organs or tissues in the human body [2]. This includes dental and orthopedic implants, ligaments, intraocular lenses, vascular grafts, artificial hearts, heart valves, biosensors, and cardiac pacemakers [5,6,7,8,9,10,11,12,13]. An implant should function flawlessly for a lifetime. As such, the development of materials that can withstand long-term implantation in the human body is a top priority, as, at present, commercially available biomaterials tend to break down after extended use due to poor biocompatibility, low fatigue strength, low corrosion and wear resistance, and a higher modulus than bone [14]. Biological biocompatibility, corrosion resistance, and mechanical biocompatibility determine the biocompatibility of orthopedic implant materials [7,15]. Biological biocompatibility examines interactions between an implant material and biological processes and includes its cancer-causing, mutational, genotoxic, or cytotoxic potential. Corrosion resistance in a biological environment is another important characteristic, as long-term orthopedic implants must be mechanically biocompatible and possess high wear resistance, high strength, and a low Young’s modulus [7]. The Young’s modulus is an important mechanical quality, as the effects of stress shielding often result in revision surgeries [15,16,17,18].

Biocompatible metals have so far been most widely used in biomedical applications. Titanium (Ti) and Ti alloys, stainless steel, and cobalt (Co) alloys are the broadly recognized biocompatible metals in the medical industry [19,20,21,22,23,24,25,26,27,28,29,30,31,32,33,34,35,36]. These materials are commonly used to replace and support fractured bone fragments as well as in dental implants, pacemaker casings, artificial heart valves, screws, plates, artificial joints, extrinsic fixators, spinal fixations, and stents [2,34,35,36].

Researchers currently prefer Ti and Ti alloys as the most advantageous biocompatible metals [19,20,21,22,23,24,25]. The utilization of Ti and Ti alloys as implant materials is deemed favorable due to their optimal properties [35]. As Ti and Ti alloys are chemically inert and possess strong fatigue resistance and a low Young’s modulus, they perform better than stainless steel and Co alloys in long-term implantation [21,37,38]. In addition, when compared to other biocompatible metals, Ti and Ti alloys exhibit significantly greater corrosion resistance [35,37]. Corrosion usually shortens the lifespan of the implants and necessitates additional surgery to replace the damaged ones.

**Figure 1:**
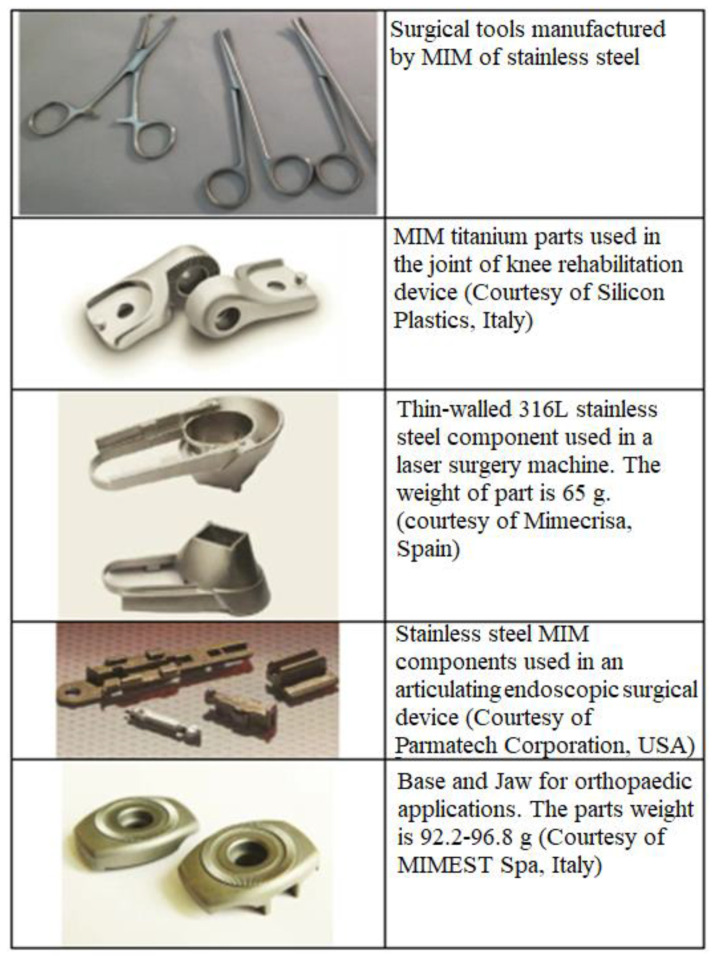
MIM-processed medical tools and equipment, reused with permission from Elsevier [60].

**Figure 2:**
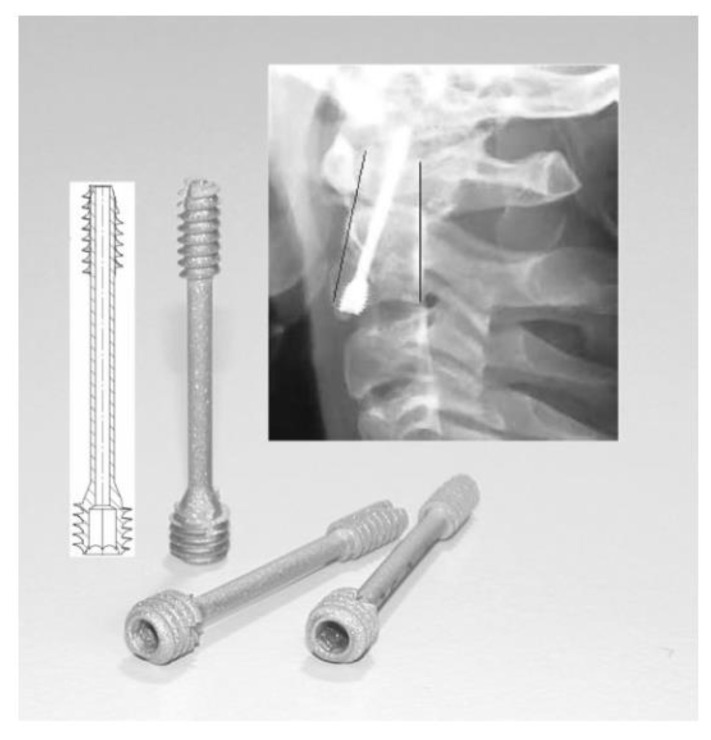
Bone screw design for MIM processing and the placement of a typical implant in the second cervical vertebra of the neck, reused with permission from John Wiley and Sons [56].

**Figure 3:**
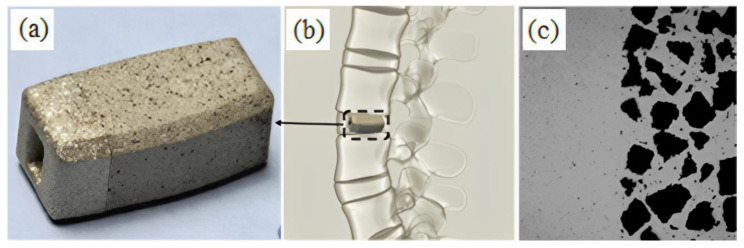
(**a**) Spinal implant of titanium produced employing the two-component-MIM process; (**b**) positioning of implant immediately after surgical procedure; and (**c**) cross-sectional microstructure exhibiting adequate joining between the dense parts and porous, reused with permission from Elsevier and John Wiley and Sons [57,60].

**Figure 4:**
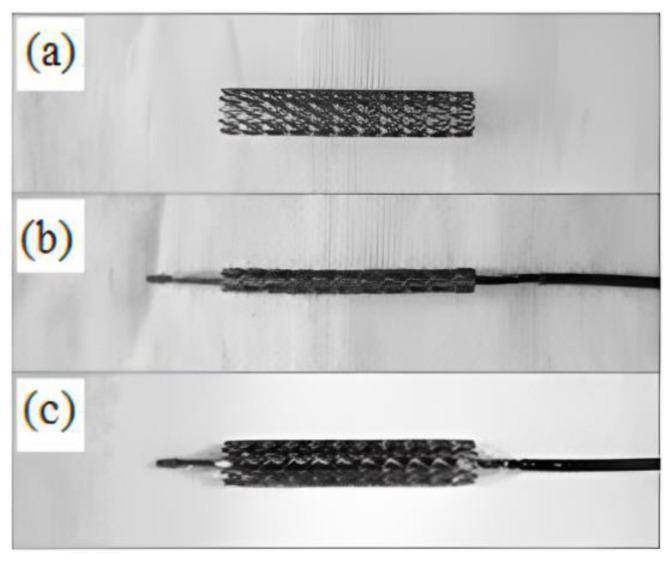
MIM stainless steel intravascular stent in (**a**) as received, (**b**) folded, and (**c**) expanded states [59].

**Figure 5:**
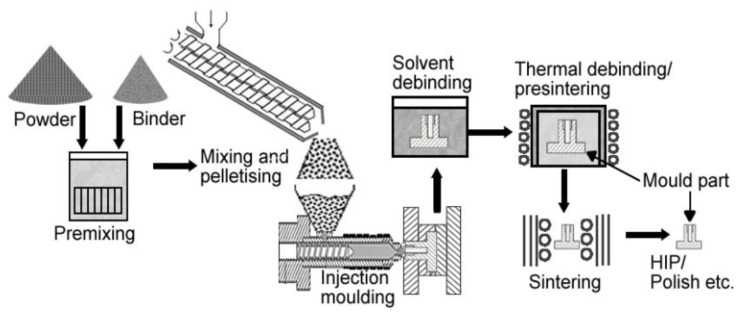
MIM processing steps [61].

Powder injection molding (PIM) is an economical method of mass-producing shaped components. Metal injection molding (MIM), a type of PIM, has made inroads into numerous industries as it is fast, cost effective, versatile, and potentially downsizable, as well as possessing outstanding design, dimensional accuracy, and mechanical properties that yield an exceptional surface finish and minimal by-products or waste [39,40,41,42,43,44,45,46,47,48]. At present, MIM is a popular method of manufacturing precise net-shaped parts for orthopedic and dental implants, surgical tools, and medical equipment [21,49,50,51,52,53,54,55]. Figure 1 depicts some commonly used MIM-fabricated medical tools and equipment. Aust et al. [56] used MIM to produce Ti-6Al-7Nb alloy bone screw implants to restore a fractured dens axis (Figure 2). Meanwhile, Barbosa et al. [57] and Barbosa [58] used two-component MIM to develop a Ti spinal implant (Figure 3). The study found that the highly porous section of the implant facilitated osseointegration via cell ingrowth and bodily fluid circulation in the interconnected pores, while the low-porosity section provided mechanical stability. Shu et al. [59] successfully fabricated MIM-based intravascular stents using 316L stainless steel powder in an effort to create stents with greater biocompatibility (Figure 4). The main steps of the production process included mixing, injection molding, debinding, and sintering (Figure 5). The binders were first mixed with the metal powder prior to undergoing the MIM process to produce a homogeneous feedstock, which was then converted into suitably sized pellets. During the MIM process, pressure and heat were used to mold the feedstock into the required shape. It was then demolded by removing the green part from the cavity of the mold. Solvent and thermal debinding techniques were used to remove the soluble and insoluble binders from the green part, respectively. Lastly, the brown debound part was sintered to produce the required mechanical and physical properties.

In the MIM process, it is highly important to get defect-free components with the desired mechanical properties. Choosing the appropriate process parameters at all stages of the MIM process is crucial to obtaining flawless components. Moreover, sintering is an indispensable step in the MIM process, and sintering parameters, especially sintering temperature, have a big impact on the mechanical properties of the components. The influence of sintering temperature on the microstructures and mechanical properties of different components is now being studied by MIM researchers from several domains [39,43,45,46].

In view of the above, as Ti and Ti alloys are superior to other existing biocompatible metals, this paper reviewed the MIM process parameters that studies published between 2013 and 2022 used to produce Ti and Ti alloy components for biomedical applications. In addition, this present study outlines the investigations of the MIM researchers over the past 10 years to attain desirable mechanical properties for Ti and Ti alloy-based biomedical components by using varying sintering temperatures.

## 2. Biomedical Applications of Titanium and Titanium Alloys

**Figure 6:**
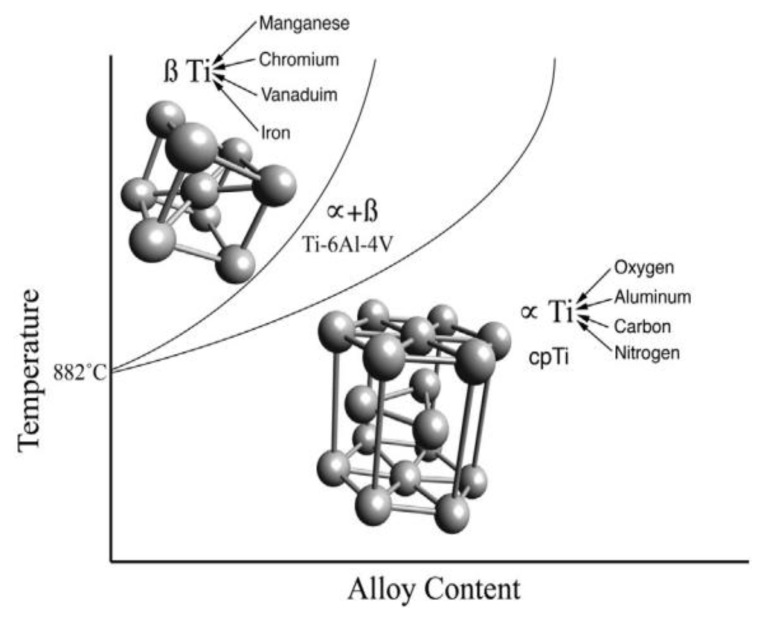
Phase of titanium alloy, reused with permission from Elsevier [81].

Ever since William Justin Kroll first discovered a method of extracting metallic Ti from its ore in the 1940s, Ti has increasingly been used in industrial and commercial applications [62,63]. Ti and Ti alloys have been extensively used in a variety of industrial fields, such as the aerospace, nuclear, marine, biomedical, automotive, and chemical industries, as they have high specific strength, low density, good biocompatibility, high fatigue strength, and good corrosion resistance [64,65,66,67,68,69,70,71,72,73,74,75]. Ti and Ti alloys provide good biocompatibility and superior corrosion performance as they naturally develop an uninterrupted, adhesive, and thin oxide film across the entire surface [76,77,78,79,80]. Figure 6 illustrates the two crystal orientations that can exist in Ti, an allotropic element. At temperatures below 882 °C, Ti remains in the alpha (α) phase with hexagonal, close-packed crystals. However, at temperatures above 882 °C, it transitions to the beta phase (β) and has body-centered cubic crystals [81,82,83]. Ti alloys with persistent α, β, and α + β phases can be produced at ambient temperature by selectively alloying them with other elements [81,84]. Aluminum (Al), carbon (C), oxygen (O), and nitrogen (N) stabilize Ti in the α phase, while manganese (Mn), chromium (Cr), iron (Fe), and vanadium (V) stabilize it in the β phase [82,85]. Hundreds of different types of Ti alloys have been developed worldwide, 20 to 30 of which, such as Ti-6Al-4V, Ti-2Al-2.5Zr, Ti-5Al-2.5Sn, Ti-Mo-Ni, Ti-32Mo, Ti-Pd, Ti-10-5-3, Ti-811, Ti-1023, Ti-1100, Ti-6242, IMI829, IMI834, BT9, and BT20, are very popular [86,87,88].

**Figure 7:**
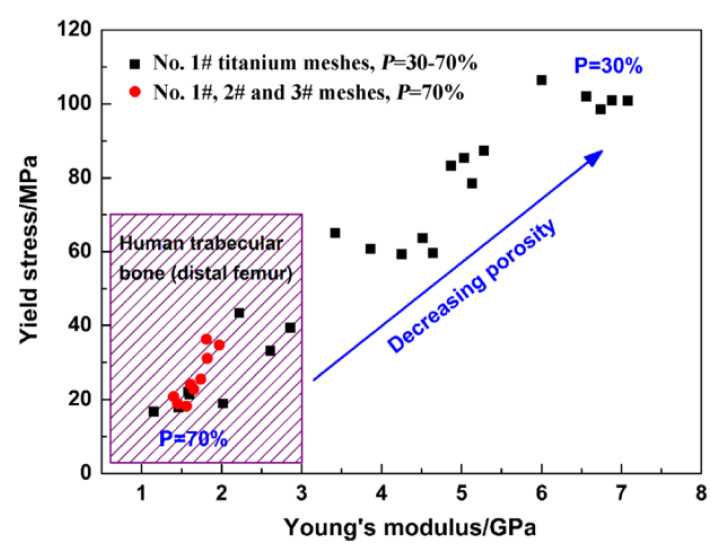
Yield stress and Young’s modulus for porous titanium compacted toward out-of-plane. Purple zones show the mechanical properties of spongy bone, reused with permission from Elsevier [106].

In the 1950s, Brånemark discovered that it was possible to permanently integrate Ti into bone. As such, they coined the term “osseointegration”, which refers to the secure fixation of Ti to bone tissues [89,90,91]. Osseointegration is commonly used to describe the biocompatible interactions that occur between a biomedical implant and the surrounding bone tissues that enable it to integrate with the bone tissues [92]. Several studies have examined the advantages of osseointegration in implants [93,94,95,96,97,98]. Sidambe [61] reported that Ti and Ti alloys have been extensively used as biomedical implants since the early 1970s. According to Manivasagam et al. [99], a combined total of GBP 2.2 million of Ti implants are transplanted into patients around the globe every year. Ti is commonly used as orthopedic and dental implants, artificial knee and hip joints, pacemakers, artificial hearts, cardiac valve prostheses, cornea backplates, bone plates, and screws for fracture fixation [61,99,100,101]. Stress shielding, which causes bone resorption, occurs when the Young’s moduli of the bone and the implant material are dissimilar [18,102,103,104]. The Young’s modulus of bone ranges between 10 and 30 GPa [18,105]. As seen in Table 1, the Young’s moduli of Ti alloys are more analogous to that of bone than other metal implant materials. Ti with a porosity of around 60% to 70% has outstanding Young’s modulus (Figure 7) and is, therefore, a promising material for bone implants [83,106]. However, the strength and porosity of a material must be balanced, as they both affect the efficacy of an implant [83,107,108].

The Ti-6Al-4V alloy, which was developed in the United States, was used for the first time in the medical industry in 1954 [2]. As Ti-6Al-4V possesses superior mechanical properties, is biocompatible, and does not interfere with innovative imaging methods such as computer tomography and magnetic resonance imaging, it remained the primary Ti alloy used in orthopedics and dentistry for a very long time [115,120,121,122,123,124,125,126,127,128,129,130]. However, multiple studies have raised the issue of the toxicity of the Al and V found in the Ti-6Al-4V alloy. Bodily exposure to Al and V has been found to cause cytotoxicity, allergic reactions, and neurological disorders [7,131,132,133,134,135]. Furthermore, although the Young’s modulus of Ti-6Al-4V (110 to 114 GPa) is lower than that of other biocompatible metals, such as stainless steel and Co alloys (Table 1), it is significantly higher than the stiffness of bone (10 to 30 GPa). Therefore, the mechanical loads of day-to-day tasks could cause stress shielding and lead to peri-prosthetic fractures, implant loosening, and bone loss [7,136,137,138,139]. As such, multiple studies have examined β-type Ti alloys to overcome the limitations of Ti-6Al-4V and other α + β-type Ti alloys [83,115,135]. According to Niinomi [140], β-type Ti alloys have low stiffness, which aids bone repair and remodeling. As β-type Ti alloys contain β-stabilizing elements, such as Nb, zirconium (Zr), molybdenum (Mo), and tantalum (Ta), their Young’s moduli are lower and closer to those of human bone, which could prevent implant loosening and bone resorption from stress-shielding. Furthermore, as they develop additional stable oxide layers, they have good biocompatibility, exceptional corrosion resistance, and are non-toxic [135,141]. As seen in Table 1, many types of β-type Ti alloys have been developed to achieve desirable Young’s modulus, tensile strength, yield strength, and elongation for biomedical applications. In this context, it is worthwhile to mention that the phase transformation process and mechanical properties of β-Ti alloys are significantly impacted by the injection molding, debinding, and sintering parameters during the MIM process.

## 3. MIM Process for Titanium and Titanium Alloys

### 3.1. Metallic Powder Selection

The attributes of a metallic powder, especially particle size and shape, substantively affect the quality of the feedstock as well as important stages of the MIM process [142,143,144,145,146,147,148,149]. According to multiple studies, metallic powder particles should possess the following characteristics to successfully complete the MIM process [39,150,151]:

- Spherical-shaped powder particles are preferred, as irregular-shaped particles cause imbalanced powder loading and more shrinkage;
- A higher packing density is required to maximally load the powder;
- Adequate interparticle friction is required to retain the shape throughout the debinding stage. Distortions are more likely to occur when larger powder particles are used, as the intraparticle contact per unit volume decreases;
- The powder particles must be non-agglomerated to produce defect-free sintered components;
- The particles should not react with the multicomponent binder system;
- The powder particles should be void-free to ensure that the sintered parts have excellent density.

As powder particle size significantly affects the MIM process, the right powder size must be chosen to produce flaw-free components. Most studies use particles that are less than 22 µm in size to improve densification, which affects the mechanical and corrosion properties of the product [2,151]. According to Piotter et al. [152], fine powder particles are preferable for metal materials; however, commercial-grade metal powders are larger than 5 µm on average. Table 2 lists the powder particle sizes of Ti and Ti alloys that MIM studies published between 2013 and 2022 used in biomedical applications.

As seen in Table 2, Ti and Ti-6Al-4V were most commonly used in MIM studies. According to Dehghan-Manshadi [179], commercially pure Ti is more commonly used in the MIM process as it is more tolerant of O, which may reach 0.4% in grade 4, than Ti-6Al-4V, which can only tolerate 0.2% O. The study found that MIM-processed, commercially pure Ti produces components with mechanical properties and chemical compositions that satisfy ASTM standards. Meanwhile, other studies have demonstrated that MIM-processed Ti-6Al-4V components have mechanical properties that are comparable to ASTM standards, especially when combined with small amounts of other elements, such as boron (B), C, gadolinium (Gd), and titanium carbide (TiC) [171,173,179,180,181,182,183,184,185,186]. As seen in Table 2, more studies have investigated the use of micro-sized powder particles in MIM than nanosized powder particles. This is because fine powder particles have a host of challenges, such as difficulty increasing the viscosity of the feedstock, difficulty attaining higher packing densities due to agglomeration, and more time needed to produce a homogeneous feedstock [39,187,188]. Furthermore, as seen in Table 2, only a few studies have examined the use of powder particles that are less than 22 µm in size. Larger particles decrease the debinding strength and increase the likelihood of distortion. Moreover, unlike smaller particles, larger particles have less intrinsic strength when they are packed together [151].

**Figure 8:**
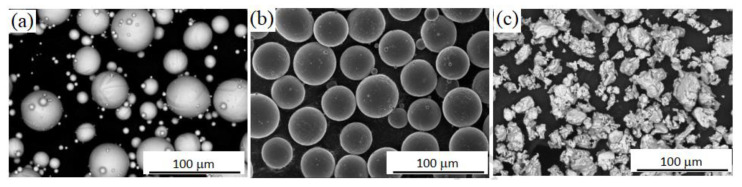
SEM images of Ti powders: (**a**) gas atomized, (**b**) advanced plasma atomized, and (**c**) HDH, reused with permission from Elsevier [179].

Powder particles are either spherical or irregular in shape, both of which possess unique properties. Spherical Ti powder particles that are less than 30 µm in size and produced using plasma atomization (from wire), gas atomization (from liquid), and plasma spheroidization (from non-spherical powder) are excellent for MIM as they possess excellent flow properties and shrink homogeneously throughout sintering [179]. Fine spherical Ti powder is expensive and has a low O tolerance, while non-spherical Ti powder costs significantly less and has a high O tolerance. As such, multiple studies have examined developing MIM procedures that can be used for hydride-dehydride (HDH) Ti powders [179,189,190,191,192,193]. Another strategy to lower the cost of MIM-fabricated components and increase the suitability of HDH powders for MIM is to combine HDH with spherical powders [193]. Figure 8 depicts the scanning electron microscopy (SEM) images of gas atomized, plasma atomized, and HDH Ti powders for MIM. As seen, the gas-atomized and advanced plasma-atomized Ti powders were spherical, while the HDH Ti powders were irregular and, therefore, a promising cost-effective alternative for MIM [68].

### 3.2. Binder Selection

A binder system is used in the MIM process to transport metal. The choice of binder is crucial, as it affects the quality of the finished product. The purpose of a binder system is to ensure that the shape of an MIM part remains unchanged until the sintering process begins as well as to help the formation during MIM. The choice of binder affects multiple variables and processes, such as packing density, powder-binder mix, feedstock flowability, injection molding, binder extraction, dimensional accuracy, and defect generation. Binders for the MIM process should possess the following characteristics [39,173,194]:

- Outstanding adhesion to Ti powder;
- Low likelihood of powder-binder separation;
- Superior wetting capabilities and flow properties;
- A viscosity that only varies slightly during MIM;
- Produce very little residual C post-burnout;
- Does not cause chemical reactions when interacting with Ti powder particles;
- Toxicity-free.

The filler phase enables the feedstock to flow easily, while the backbone polymer serves as a second component that helps retain the shape of the sample. Additives, such as lubricants, surfactants, and dispersants, are commonly used as a final binder to improve the powder-binder interface. Table 3 lists the binders that MIM studies published between 2013 and 2022 used.

As seen in Table 3, most of these studies examined the use of paraffin wax for feedstock flowability, polyethylene as the backbone polymer for Ti and Ti alloys in biomedical applications, and stearic acid as a surfactant as it promotes effective powder-binder adhesion. More recent MIM studies, however, examined the use of palm stearin as a binder. Iriany et al. [196] were the first to examine the use of a palm stearin binder to circumvent debinding, which is the most time-consuming stage of the MIM process. The ability of this binder to serve as both a lubricant and a surfactant is a very important property [176,197]. Indonesia, Malaysia, Nigeria, and Colombia, as well as other African, Latin American, and Asian nations, are directly involved in palm oil production. This has made palm stearin more accessible to end users as a cost-effective commercial binder. Furthermore, as a palm stearin binder poses few environmental hazards, it is highly recommended for use in MIM [198].

### 3.3. Preparation of Feedstock

Ti and Ti alloys are typically mixed with binders to produce feedstocks. Powder loading and binder type significantly affect the characteristics of the produced feedstock. Optimal powder loading, based on critical powder loading, is recommended when using MIM to fabricate Ti and Ti alloy-based biomedical components. The powder particles must be in void-free contact with one another for critical powder loading [199,200]. Unlike critical powder loading, optimal powder loading uses fewer powder particles, significantly decreases feedstock viscosity, greatly simplifies the MIM process, and produces products with excellent mechanical properties and only minor distortions [201]. Higher powder loadings complicate the mixing process and increase feedstock viscosity [202]. On the other hand, higher binder content and insufficient powder loading in the feedstock cause higher shrinkage in sintered components. Lower powder content significantly affects the density of sintered components [203].

Powder particle size significantly affects critical powder loading during MIM. Smaller powder particles have a larger surface area, which lowers critical powder loading [204]. As such, many studies have examined multiple methods of determining the critical powder loading for MIM. Aggarwal et al. [205] used a torque rheometer to calculate the critical powder loading based on the rheological behavior of the feedstock. The powder loading range at which dramatic transformations in rheological parameters, such as viscosity, power law exponent, and flow activation energy, occurred was designated as the critical solid loading. Kong et al. [206] used a twin-screw mixer to gradually increase the volume of powder in the binder and a different strategy to determine the critical powder loading. The mixing torque was found to increase drastically when the critical powder loading was at a specific level. Mutsuddy and Ford [207], as well as Reddy et al. [208], gradually added an oil binder to a powder to obtain a critical powder volume concentration. The incremental addition of oil caused the torque to initially increase sharply before gradually decreasing until it reached a stable state. The critical powder loading was determined to be when the torque increased to its highest. Optimal powder loading is critical to producing defect-free MIM-processed Ti and Ti alloy components. This can be achieved by using powder loadings that are 2 to 5 vol.% less than the critical powder loading [199].

**Figure 9:**
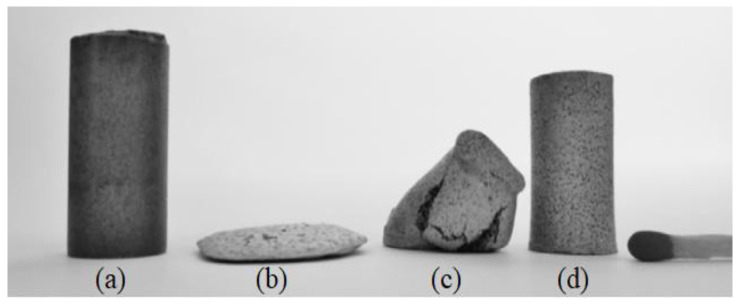
MIM samples: (**a**) unsintered with powder loading 72 vol.%; (**b**–**d**) sintered at 1200 °C for a period of 3 h with powder loading 72, 75, and 80 vol.%, reused with permission from Elsevier [73].

As seen in Table 4, most MIM studies used powder loadings of 50 and 67 vol.% for Ti and Ti alloys, while others used powder loadings of 70 vol.% and higher. De Freitas Daudt et al. [73] produced extremely porous MIM-processed Ti and reported that it was difficult to obtain open surface porosity and to retain the shape during debinding and sintering. The study examined the use of three different powder loadings: 72, 75, and 80 vol.%, and found that MIM components with 72 vol.% powder loading entirely collapsed (Figure 9b), 75 vol.% yielded less shape deformation (Figure 9c), and 80 vol.% powder loading yielded the best shape retention post-thermal debinding and sintering (Figure 9d).

### 3.4. Rheological Properties

**Figure 10:**
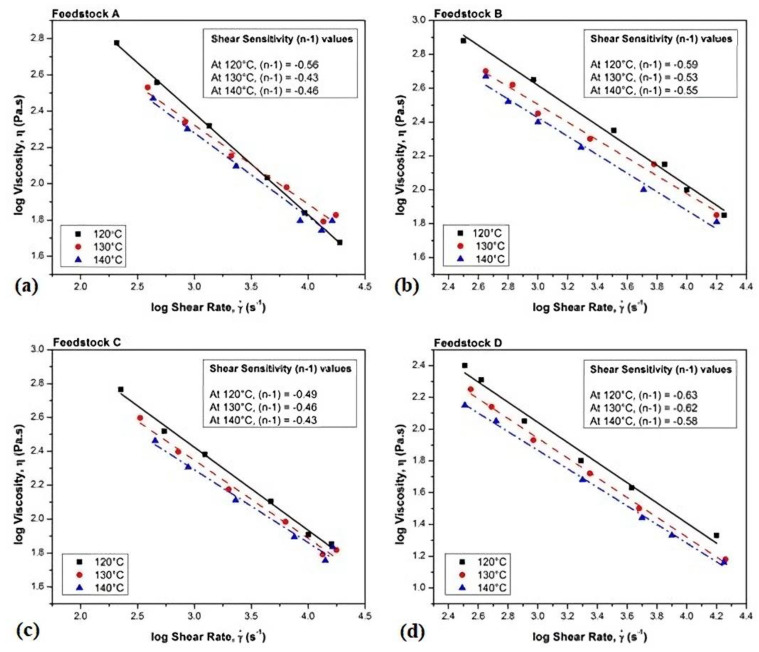
Viscosity versus shear rate at three different temperatures for (**a**) Feedstock A, (**b**) Feedstock B, (**c**) Feedstock C, and (**d**) Feedstock D, reused with permission from Elsevier [157].

The rheological characteristics of the feedstock, which closely correlate with viscosity, shear rate, and shear stress, can be analyzed to optimize the injection molding process. A capillary rheometer is typically used to measure rheological properties. At a specific molding temperature, a shear rate of 10^2^ to 10^5^ s^−1^ and a viscosity of less than 1000 Pa·s are best for Ti- and Ti alloy-based feedstocks to flow into the mold cavity [19,176,199]. The different rheological behaviors that a fluid exhibits when flowing are a critical factor. When shear thickening or dilatant flow occurs, the viscosity increases as the shear rate increases, which increases the likelihood of powder-binder segregation during MIM [200]. Conversely, when shear thinning or a pseudo-plastic flow occurs, the viscosity decreases as the shear rate increases, which is desirable for MIM as it helps fill the mold cavity effectively and faultlessly as well as maintain the shape of MIM parts [68,151,175,199]. Hayat et al. [157] produced MIM-processed Ti components using pure Ti powder by ensuring that the formulated feedstocks exhibited pseudo-plastic behavior during rheological analysis (Figure 10). According to Thavanayagam et al. [147], as some feedstocks that exhibit pseudo-plastic behavior may also exhibit dilatant flow behavior when subjected to high shear rates, it is necessary to thoroughly evaluate the rheological properties across a range of shear rates.

Equation (1) depicts the interdependence of viscosity η and shear rate Υ in non-Newtonian fluids [210]:

(1) $$\eta =K\Upsilon^{n-1}$$

where K is a constant and n is the flow behavior index. In general, n provides a clear understanding of the shear sensitivity and flow behavior of the powder-binder mixture. When n > 1, the feedstock exhibits dilatant behavior. Conversely, the feedstock exhibits pseudo-plastic behavior when 𝑛 < 1 [176,177,210]. Temperature-dependent viscosity is another significant factor affecting the rheological behavior of a powder-binder mixture. Equation (2) depicts the flow activation energy E, which correlates with the Arrhenius equation and, generally, depicts how temperature affects feedstock viscosity [211]:

(2) $$\eta \left(T\right)=\eta_{o}\exp\;\left(E/RT\right)$$

where $\eta_{o}$, R, and T are the viscosities at the reference temperature, gas constant, and temperature, respectively. E can be obtained from the slope of the plot of ln⁡(η) against 1/T. The smaller the E, the less susceptible the viscosity was to temperature changes, which prevented unpredictable flows throughout the MIM [202,212].

Rheological measurement is a pivotal step in MIM. Multiple studies have analyzed the rheology of Ti and Ti alloy feedstocks, with most of them conducting MIM with feedstocks that exhibit pseudo-plastic behavior [19,154,159,176,177]. An optimal powder loading and a feedstock that exhibits pseudo-plastic behavior could produce crack-free components.

### 3.5. Metal Injection Molding Process

The second step of MIM is to mold the prepared Ti and Ti alloy feedstocks into the desired shape. Molding parameters significantly affect the characteristics of the injected pieces, with defects frequently occurring during MIM. Defects that emerge during the final phases of MIM typically cannot be eliminated. Short shots, flashing, jetting, powder-binder segregation, and cracks are the most common defects that occur during MIM. While some of these flaws are easily observable, others can only be found during debinding and sintering. However, proper injection molding parameters, such as injection pressure, holding pressure, melt and mold temperature, and injection duration, can be used to correct these defects.

**Figure 11:**
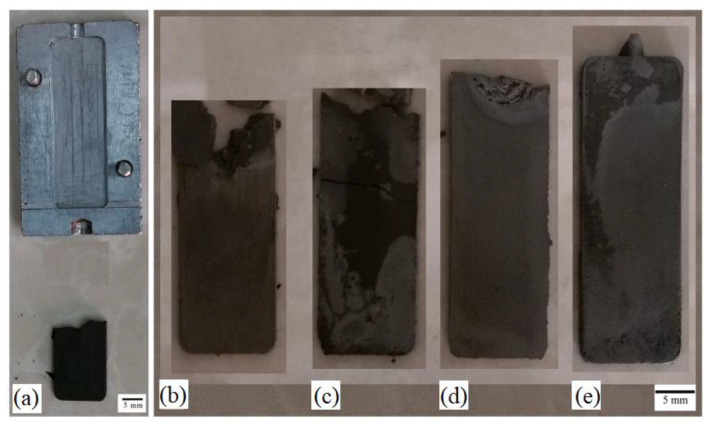
Green parts with powder loading of: (**a**) 60 vol.% at 165 °C; (**b**) 53 vol.% at 150 °C; (**c**) 53 vol.% at 155 °C; (**d**) 53 vol.% at 160 °C; and (**e**) 53 vol.% at 165 °C. Reused with permission from Elsevier [68].

**Figure 12:**
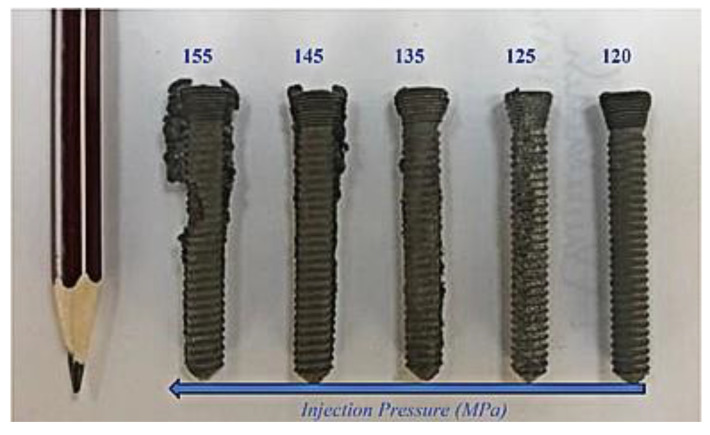
Injection-molded components in high-pressure applications [19].

In the field of injection molding, a short shot, a defect that occurs when the powder loading, mold temperature, and injection pressure are incorrect, is a potential hindrance. Moghadam et al. [68] observed short-shot defects in injection-molded Ti components (Figure 11). As seen in Figure 11a–e, although the green parts were manufactured using injection processes at temperatures of 150 to 165 °C, short shot defects were only prevented when a feedstock temperature and powder loading of 165 °C and 53 vol.%, respectively, were used. As such, the study examined the best method of regulating feedstock temperature and powder loading to produce flaw-free components. Flashing is another common defect that occurs during injection molding. Urtekin et al. [19] produced MIM-processed cortical-bone screws with Ti-6Al-4V powders and binders and reported that the green component maintained its geometry at a pressure of 120 MPa (Figure 12). As seen in Figure 12, challenges, including ejection from mold and flashing, occurred when the pressure exceeded 120 MPa. These issues were believed to arise due to high injection pressures. Therefore, flash-free components could be produced by using the correct injection pressure and properly clamping the mold during injection molding.

In summary, it is crucial to obtain defect-free green Ti and Ti alloy components during injection molding. As such, the injection molding parameters should be correctly adjusted to produce flawless parts for debinding and sintering.

### 3.6. Debinding Process

**Figure 13:**
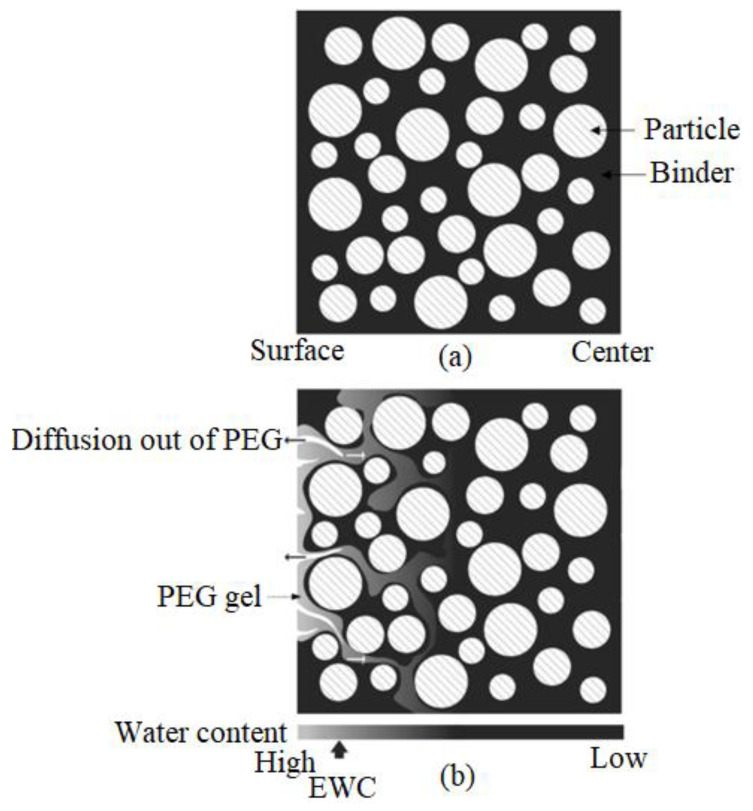
Schematic diagrams demonstrating the distributions of binder in (**a**) as molded and (**b**) preliminary stages of solvent debinding on the basis of water extraction, reused with permission from Elsevier [213].

**Figure 14:**
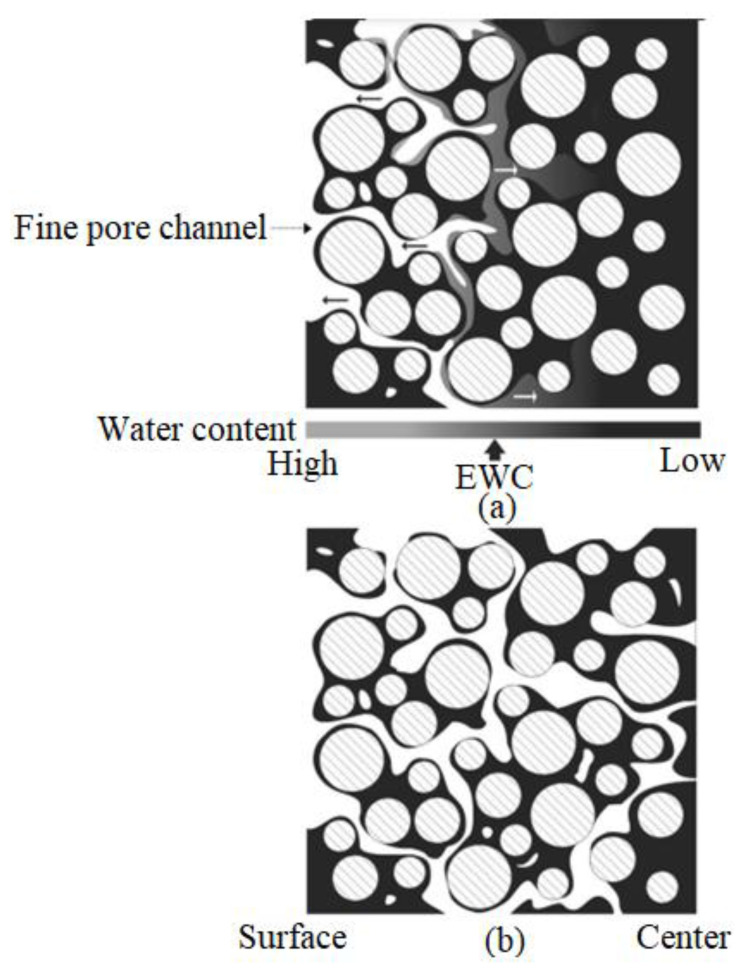
Schematic diagrams demonstrating the distributions of binder in (**a**) intermediary and (**b**) final stages of solvent debinding on the basis of water extraction, reused with permission from Elsevier [213].

Solvent and thermal debinding are debinding methods that are frequently used in MIM studies. Solvent debinding, which involves immersing injection-molded components in a liquid solvent and conducting the procedure at a specific temperature, can be used to extract soluble binders from a multi-component binder system [39,73,170]. Diffusion is commonly used to eliminate the soluble binder. Yang et al. [213] schematically outline the various stages of the solvent extraction process (Figure 13 and Figure 14). As seen in Figure 13 and Figure 14, the diffusion of solvent molecules into the binder marks the beginning of the solvent extraction process, which causes a swelling gel to form. The soluble binder dissolves into the solution due to sufficiently potent interactions between the solvent and the soluble binder that counteract the intermolecular forces [214]. The pore size increases as the insoluble binder is kept in the contact area. This open-pore structure accelerates the thermal extraction process and facilitates the quick removal of the insoluble binder [215]. Table 5 lists the parameters that extant studies have used to solvent debind a variety of injection-molded Ti and Ti alloy components.

**Figure 15:**
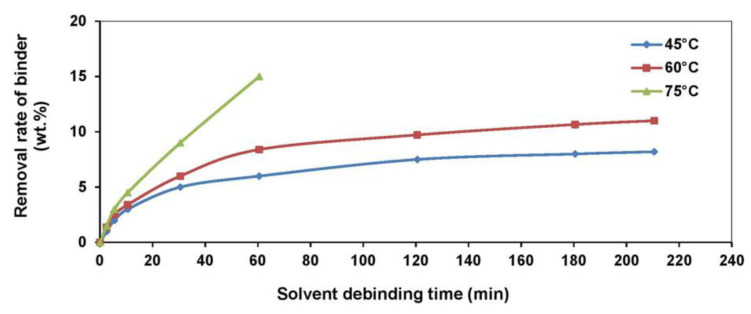
Elimination of soluble binder during the process of solvent debinding at different temperatures, reused with permission from Elsevier [68].

As seen in Table 5, solvent debinding was mostly conducted at temperatures between 40 and 60 °C, and heptane and hexane were the preferred choices of solvent. Temperature is crucial in solvent debinding, as higher temperatures have been found to improve soluble binder and solvent interactions by transforming the solubility and the binder diffusion coefficient [216]. Often, very little soluble binder remains in components after solvent debinding. During the thermal debinding process, this can be eliminated along with the insoluble binder. It is important to produce solvent-debound components that are crack-free. As seen in Table 5, Moghadam et al. [68] examined the use of three different solvent debinding temperatures of 45, 60, and 75 °C on injection-molded Ti components or green parts. Although a temperature of 75 °C removed the binder very quickly, the solvent debinding process was halted immediately after 60 min as cracks began to form (Figure 15). This is believed to have occurred due to the extremely rapid extraction rate and the potential softening of the low-density polyethylene backbone. Components that were solvent debound at 45 and 60 °C were defect-free. These results indicate the importance of debinding temperature in solvent debinding. Therefore, defect-free solvent debound components can be produced by using the correct solvent type, solvent debinding temperature, and time.

Thermal debinding is commonly used to eliminate insoluble binders from injection-molded components. In the early stages of thermal debinding, the binder degradation rate determines how quickly the binder flows from inside the component to its surface. As the polymer degradation process progresses, pores near the surface open and help dispel the burnout gases of the binder via interconnected pore channels [199,217]. Due to polymer losses during thermal debinding, the component typically weakens immediately. As such, thermally debound components must be handled with care. According to Hamidi et al. [2], thermally debound components experience thermal, gravitational, and residual stresses, which could cause defects or cracks to form as the polymer degrades. Furthermore, the subsequent sintering process will aggravate the microscopic defects that formed during thermal debinding. As such, the thermal debinding schedule must be designed properly to overcome these issues. Table 6 lists the parameters that extant studies have used to thermally debind Ti and Ti alloy components. As seen in Table 6, temperatures between 500 and 700 °C were the best for the given thermal extraction durations.

### 3.7. Sintering Process

Sintering, the last stage of the MIM process, transforms debound components into a robust mass and significantly affects the physical and mechanical properties of the produced component. The sintering temperatures are typically 70 to 90% of the melting temperature of the metal [2,218]. Vacuum, air, argon, hydrogen, and reduced nitrogen/hydrogen atmospheres are commonly used during sintering. The exterior surface of the powder particles undergoes remarkable chemical changes and atomic diffusion when the sintering temperature increases from one-half to two-thirds of the melting point of the metal. Thermolysis occurs as the temperature increases, which burns out the remaining binders [2,39]. Sintering uses several mechanisms, such as lattice diffusion, surface diffusion, grain boundary diffusion, plastic flow, evaporation-condensation, and viscous flow, for mass transport [219]. These mechanisms encourage the growth of necks between particles, which improve the strength of the consolidated powders [39,150]. Table 7 lists the parameters that extant studies published between 2013 and 2022 have used to sinter Ti and Ti alloy components. As seen in Table 7, a temperature range of 1100 to 1500 °C, a heating rate of 3 to 10 °C/min, a duration of 1 to 8 h, and a vacuum or argon atmosphere were preferred.

**Figure 16:**
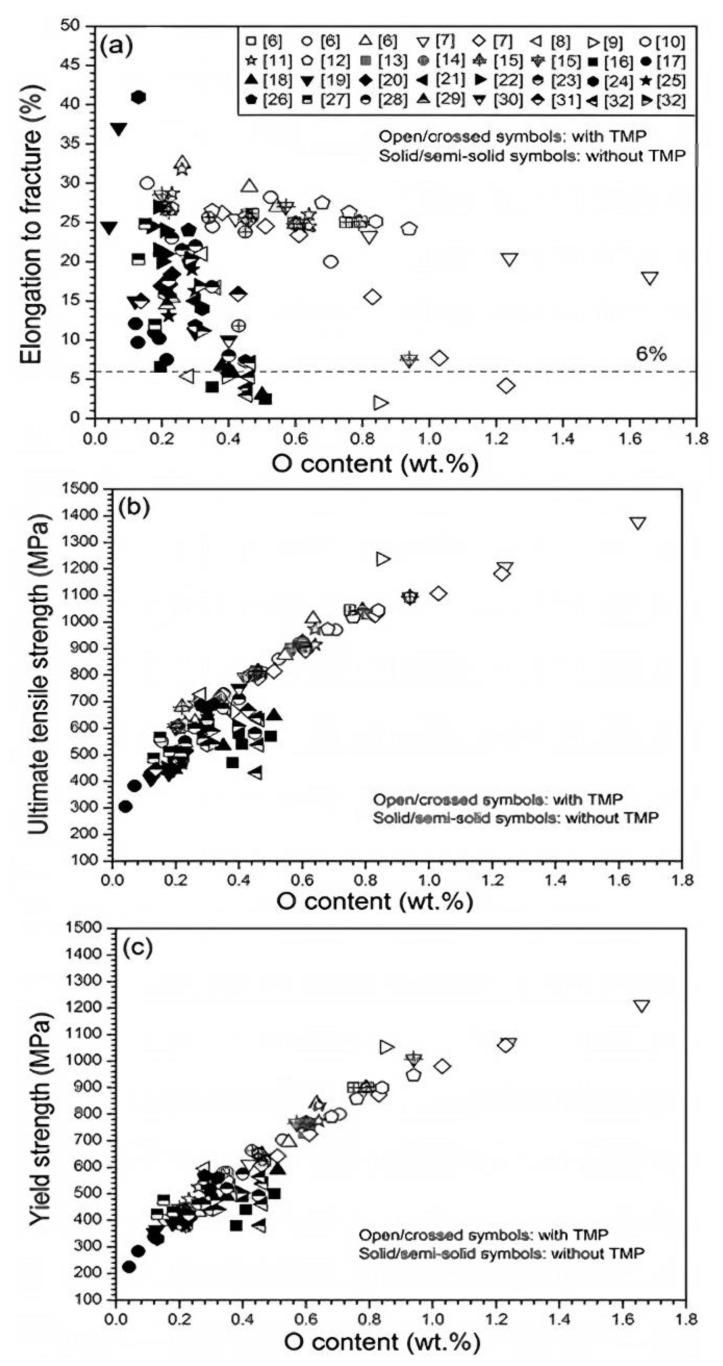
Tensile properties of unalloyed titanium versus O content: (**a**) elongation to fracture, (**b**) ultimate tensile strength, and (**c**) yield strength. Samples were produced through powder metallurgy, MIM, and additive manufacturing routes and reused with permission from Elsevier [220].

It is important to mention that Ti and Ti alloys have a profound affinity for impurities, notably O and C [193]. O reduces the stress corrosion resistance, fatigue strength, and tensile ductility of the MIM-processed sintered Ti and Ti alloy components [179]. According to Luo et al. [220], the ultimate tensile strength and yield strength of the unalloyed Ti increased, but tensile ductility decreased as the O content increased (Figure 16). The initial powder, binder system, and sintering environment are the primary contributors to O contamination in the MIM process. Usually, with careful selection of powder and binder as well as precise control over the injection, debinding, and sintering processes, O content can be kept lower than 0.3 wt.% for commercially pure Ti components [179]. On the other hand, in order to prevent the development of TiC in structure, which could reduce corrosion resistance, weaken the elongation and fatigue properties, and increase the Young’s modulus of sintered parts, the C content must be kept to less than 0.08 wt.% [221].

During the MIM process, it is essential that sintered components exhibit the desired mechanical properties. Mechanical properties are significantly influenced by sintering parameters, particularly sintering temperature. Table 8 summarizes the mechanical properties of the Ti and Ti alloy components that sintered at different temperatures between 2013 and 2022.

**Figure 17:**
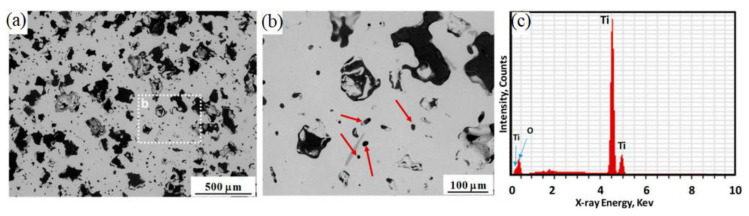
(**a**,**b**) SEM image of titanium sintered at 1250 °C. The micron-size pores are indicated by arrows, and (**c**) energy-dispersive X-ray spectroscopy (EDS) analysis of the scaffold [156].

Based on Table 8, a sintering temperature range of 1100 to 1500 °C was employed by the researchers to obtain a Young’s modulus of 7.80 to 103 GPa and a tensile strength of 395 to 1154 MPa. Dehghan-Manshadi et al. [156] examined the optimal MIM parameters for porous Ti scaffolds. Figure 17 depicts the SEM micrographs of components sintered at 1250 °C. However, the sintered components contained a few big and irregular-shaped pores that were 150 to 200 µm in size (Figure 17a) as well as microsized pores (Figure 17b) due to the elimination of the space holder and binder as well as the sintering of the Ti particles. The optimal pore size for body fluid transportation and mineralized bone growth ranges between 100 and 300 µm [222,223]. The study found that the rough interior walls of the big pores were better suited for new bone tissue ingrowth. An EDS of the sintered components revealed that the microstructure did not contain chlorine or potassium (Figure 17c); therefore, all the space holders had been completely removed during water immersion. Furthermore, components that had been sintered at 1250 °C had a Young’s modulus of 7.80 GPa. The Young’s modulus increased to 22.0 GPa at 1300 °C when the porosity decreased. Bootchai et al. [160] found that MIM-processed Ti components sintered at 1150 °C had a Young’s modulus and tensile strength of 99 GPa and 542 MPa, respectively. A close tensile strength of 617 MPa was obtained by Hayat et al. [157], who sintered Ti components at 1300 °C.

**Figure 18:**
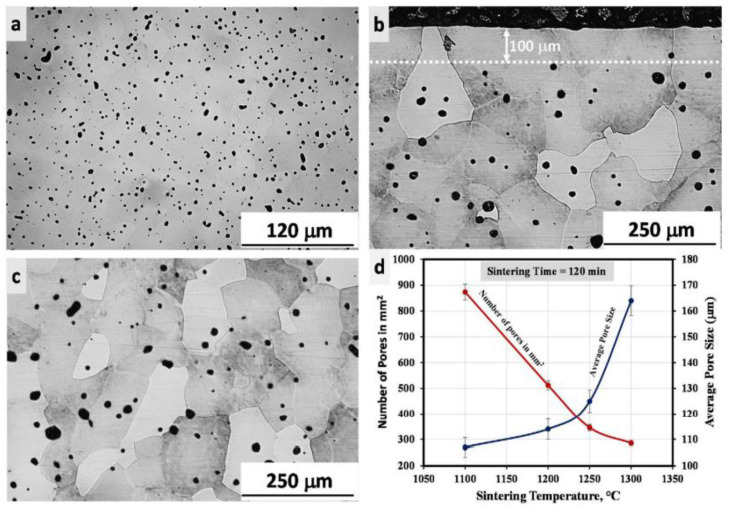
(**a**) Specimen surface after polishing that was sintered at 1250 °C for 2 h; (**b**) surface microstructure of the similar specimen; (**c**) at the center of the specimen; and (**d**) number of pores and average pore size as a function of sintering temperature, reused with permission from Elsevier [46].

Dehghan-Manshadi et al. [46] identified the best processing parameters to fabricate MIM-processed HDH Ti with minor deformations. Spherically dispersed and small-sized pores were observed in the sintered sample (Figure 18a). A closer examination revealed that an almost non-porous, thin surface layer had formed (Figure 18b) in contrast to the central section of the component (Figure 18c). As seen in Figure 18d, the number of pores significantly decreased and the average pore size increased as the sintering temperature increased. Furthermore, changes in pore size and distribution due to increasing the sintering temperature may directly affect the mechanical properties of sintered components. Components sintered at 1250 °C had a tensile strength and elongation of 395 MPa and 12.5%, respectively.

**Figure 19:**
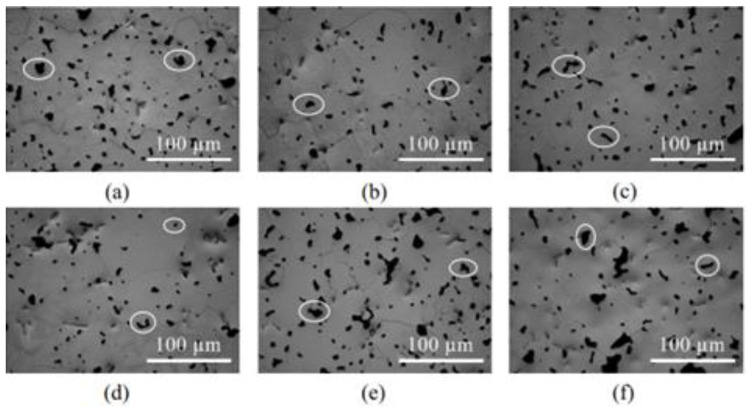
Optical micrographs of (**a**) Ti-8Mn, (**b**) Ti-9Mn, (**c**) Ti-12Mn, (**d**) Ti-13Mn, (**e**) Ti-15Mn, and (**f**) Ti-17Mn. Some pores are illustrated by ellipses, reused with permission from Elsevier [165].

**Figure 20:**
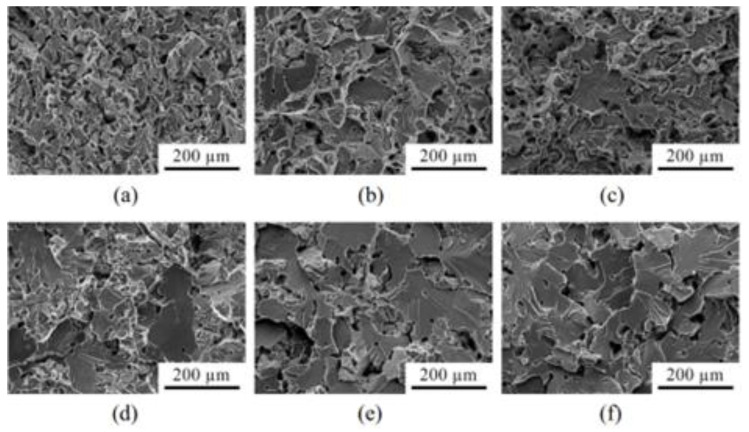
SEM fractographs of tensile-tested samples: (**a**) Ti-8Mn, (**b**) Ti-9Mn, (**c**) Ti-12Mn, (**d**) Ti-13Mn, (**e**) Ti-15Mn, and (**f**) Ti-17Mn, reused with permission from Elsevier [165].

As a prospective low-cost material, Santos et al. [165] fabricated Ti-Mn alloy components through MIM that had been sintered at 1100 °C for biomedical applications. Figure 19 depicts the optical micrographs of Ti-Mn alloys containing 8 to 17 mass% of Mn. The average grain diameter, which was 69.8 ± 6.1 μm, did not significantly differ between the alloys. As seen in Figure 19, each alloy contained large, interconnected pores and small, closed pores, with most of the pores (ellipses) located at the grain boundaries. An evaluation of the mechanical properties of the examined alloys indicated that the Ti-9Mn alloy had the best combination of tensile strength (1046 MPa) and elongation (4.7%). Figure 20 depicts the SEM fractographs of the tensile-tested components. As seen in Figure 20a–c, the Ti-9Mn and Ti-12Mn alloys had larger regions of concentrated cleavage-like structures and dimples than the Ti-8Mn alloy. Figure 20e,f depict the unique massive trans-granular cleavage-like structures found in the Ti-15Mn and Ti-17Mn alloys. The study also found that the Ti-8Mn to Ti-17Mn alloys had higher Young’s moduli. The Young’s moduli of the Ti-8Mn, Ti-9Mn, Ti-12Mn, Ti-13Mn, Ti-15Mn, and Ti-17Mn alloys were 87, 89, 96, 99, 98, and 103 GPa, respectively.

**Figure 21:**
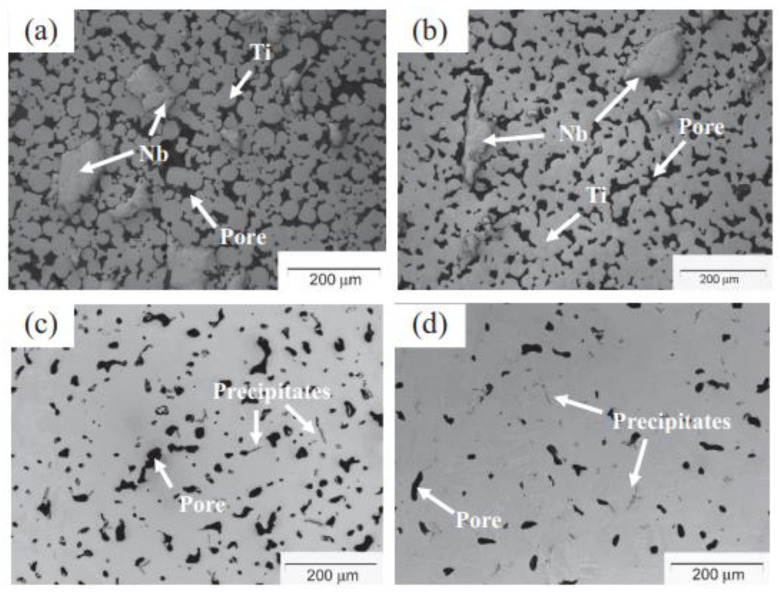
Optical micrographs of Ti-16Nb samples sintered at (**a**) 900 °C, (**b**) 1100 °C, (**c**) 1300 °C, and (**d**) 1500 °C, reused with permission from Elsevier [167].

The mechanical properties of Ti-Nb alloys make them viable bioactive implants. Zhao et al. [167] fabricated MIM-processed Ti-16Nb alloys and observed microstructural changes in components sintered at four different temperatures: 900, 1100, 1300, and 1500 °C (Figure 21). As seen in Figure 21, the component sintered at 900 °C contained Ti and Nb particles that matched their primeval particle morphologies. Although Ti particle diffusion had begun, boundaries were clearly visible between the Ti and Nb particles. It was difficult to observe the Ti particle boundaries in the Ti-16Nb alloy sintered at 1100 °C as it mostly contained irregular-shaped residual pores. Unlike Ti-16Nb alloys sintered at 900 and 1100 °C, the removal of observable Nb particles and the existence of thin needle-shaped precipitates were the most clearly observable microstructural characteristics in Ti-16Nb alloys sintered at 1300 and 1500 °C. The porosity was also found to decrease from roughly 25 to 6% as the sintering temperature increased. The ultimate tensile strength and the Young’s modulus of the Ti-16Nb alloy sintered at 1500 °C were 667 MPa and 80 GPa, respectively. Bidaux et al. [169] sintered Ti-17Nb alloys at 1400 °C that had a Young’s modulus of 76 GPa. In comparison to Zhao et al. [167] and Bidaux et al. [169], a higher Young’s modulus of 100 GPa was obtained by Yılmaz et al. [168], who carried out the sintering of MIM-processed Ti-Nb alloys at 1500 °C.

**Figure 22:**
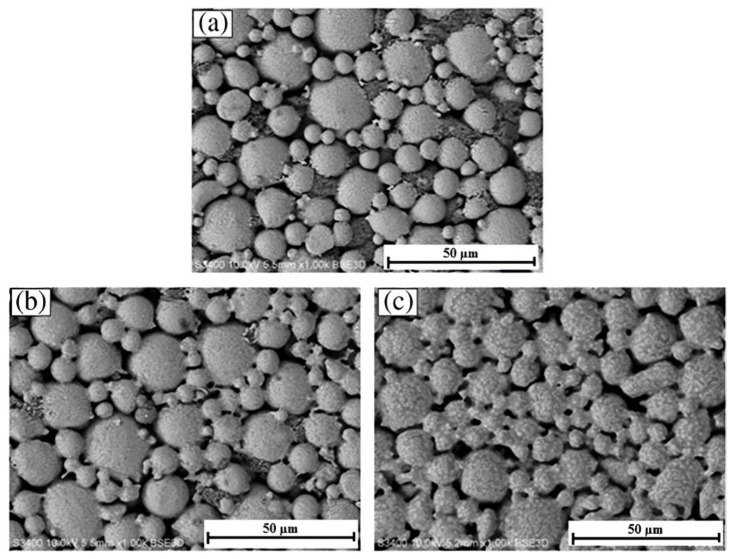
Surface morphology of Ti-6Al-4V/HA composites sintered at (**a**) 1100 °C, (**b**) 1200 °C, and (**c**) 1300 °C, reused with permission from Elsevier [176].

Nor et al. [174] fabricated Ti-6Al-4V components on the grounds of MIM. The optimum sintering temperature was determined to be 1200 °C. Ti-6Al-4V alloys sintered at 1200 °C had tensile strengths of 934.33 MPa. Holm et al. [171] fabricated Ti-6Al-4V components with Gd powder and sintered them at 1350 °C. Adding Gd was found to increase the porosity of the Ti-6Al-4V alloys from 3.6 to 5%. However, the addition of 1 wt.% of Gd decreased tensile strength from 824 to 749 MPa. Therefore, Ti-6Al-4V + Gd components had higher porosity but lower tensile strength than Ti-6Al-4V components. Arifin et al. [176] examined the viability of fabricating PIM-processed Ti-6Al-4V and hydroxyapatite (HA) composites sintered at 1100, 1200, and 1300 °C. Figure 22 depicts the components sintered at different temperatures. As seen in Figure 22, residual HA particles were detected between the gaps of the Ti particles, and the Ti particles were covered in partially decomposed HA. Some HA groups were observed on the surface at 1100 °C; however, they were not detected when the sintering temperature increased significantly. The presence of Ti atoms accelerates the formation of titanium oxide (TiO_2_) and HA dehydroxylation [224,225]. Inter-diffusion occurred at the Ti-HA interface due to the migration of HA towards Ti bulk and Ti atoms to O and HA atoms. As O is an interstitial atom, it diffuses into the Ti lattice until saturation. The Ti was then oxidized, which slowed O diffusion. Composites sintered at 1300 °C had a Young’s modulus of 44.26 GPa, which is very close to that of human bone. Ramli et al. [177] reported that Ti-6Al-4V and bioactive wollastonite (WA) were excellent for implantation applications. The PIM-processed Ti6Al4V/WA composites sintered at 1100, 1200, and 1300 °C had Young’s moduli of 18.10, 15.62, and 14.57 GPa, respectively.

Kafkas and Ebel [113] fabricated Ti-24Nb-4Zr-8Sn components based on MIM. Components sintered at 1400 °C yielded the highest tensile strength (656 MPa) and Young’s modulus (54 GPa). Increasing the sintering temperature from 1400 to 1500 °C did not significantly affect strength. On the other hand, Suwanpreecha et al. [55] fabricated novel Ti-27.5Nb-8.5Ta-3.5Mo-2.5Zr-5Sn components sintered at 1000 to 1400 °C. Sintering at 1000 °C did not adequately sinter the components, while sintering at 1100 °C yielded the best tensile strength (1154 MPa) and the highest elastic modulus (98 GPa).

## 4. Concluding Remarks and Future Directions

MIM is an exemplary manufacturing approach for fabricating small and intricate components for the biomedical industry at a low cost. This present study reviewed the MIM parameters that extant studies have used to fabricate Ti and Ti alloy biomedical products over the past 10 years. Ti and Ti alloys outperform other biocompatible metals, such as stainless steel and Co alloys, in long-term implantation due to their low Young’s modulus, strong fatigue resistance, and chemical inertness. The drawbacks of the Ti-6Al-4V and other α + β-type Ti alloys have been mostly resolved by β-type Ti alloys. Due to containing β-stabilizing elements such as Zr, Nb, Mo, and Ta, the Young’s moduli of β-type Ti alloys are closer to those of human bone, which could impede the loosening of implants and the resorption of bone from stress-shielding. Moreover, Ti with a porosity between 60 and 70% is regarded as a viable material for bone implants due to its remarkable Young’s modulus. In this review paper, we acknowledge the extensive usage of Ti and Ti alloys as biomedical implants.

A crucial step in the MIM process is choosing the appropriate powder particle size. Although most studies used microsized instead of nanosized powder particles for Ti and Ti alloys, further research using fine particles could be useful to comprehend the impact of such particles on different stages of the MIM process. When preparing feedstock, the majority of researchers chose to employ high powder loading, but this often makes the process of mixing powder and binder challenging and is responsible for the incomplete filling of the mold cavity during the injection molding process. Therefore, it is recommended to use optimal powder loading. The MIM process is dramatically streamlined by optimal powder loading since it requires fewer powder particles compared to critical powder loading, substantially reduces the viscosity of feedstock, and yields products with superior mechanical properties and minimal deformations. Cracks, short shots, jetting, and flashing are common defects for injection-molded components. Some of these imperfections can be seen instantly, but others are only revealed during the debinding and sintering processes. Defects that arise during the injection process can be avoided by selecting the injection molding parameters appropriately. The debinding process is known to be a sensitive stage, as parts remain extremely fragile during this period. It is preferable not to employ an excessively high solvent debinding temperature during the solvent debinding process to achieve crack-free components. This review paper demonstrated the competence of MIM researchers to produce sintered Ti and Ti alloy biomedical components. Basically, the sintering temperature has an immense effect on the mechanical properties of the sintered components. Finally, a doorway into the future of biomedical applications may be opened by the production of multi-functional biomedical products using Ti and Ti alloy materials along with other metal- or ceramic-based materials through the two-component metal injection molding (2C-MIM) process.

## Figures and Tables

**Table 1:** Comparison of the mechanical properties of implant materials.

Material	Young’s Modulus (GPa)	Tensile Strength (MPa)	Yield Strength (MPa)	Elongation (%)	References
**Stainless steels and Co-based alloys**					
316L stainless steel	200	500–1350	200–700	10–40	[2,18,60,83,109,110,111,112,113,114,115,116,117,118,119]
Co-Cr	200	–	500	8	
Co-Ni-Cr	220	–	850	20	
**Type of alloy: α**					
Pure Ti grade 1	103	240	170	24	
Pure Ti grade 2	103	345	275	20	
Pure Ti grade 3	103	450	380	18	
Pure Ti grade 4	104	550	485	15	
**Type of alloy: α + β**					
Ti-6Al-4V	110–114	895–930	860	6–10	
Ti-6Al-7Nb	114	900–1050	880–950	8.1–15	
Ti–5Al-2.5Fe	110	1020	780	6	
Ti-3Al-2.5V	100	585	690	15	
**Type of alloy: β**					
Ti-13Nb-13Zr	79–84	973–1037	836–908	10–16	
Ti-35Nb-7Zr-5Ta	55	590	800	20	
Ti-35Nb-7Zr-5Ta-0.35O	41–46	876–1015	864–966	2.2–15.6	
Ti-29Nb-13Ta-4.6Zr	80	911	864	13.2	
Ti-35.3Nb-5.1Ta-7.1Zr	55	597	547	19	
Ti-24Nb-4Zr-8Sn	54–62	655–720	627–685	2–9	
Ti-12Mo-6Zr-2Fe	74–85	1060–1100	100–1060	18–22	
Ti-15Mo-5Zr-3Al	80	852–1100	838–1060	18–25	

**Table 2:** Particle sizes of titanium and its alloys used in MIM (years: 2013–2022).

Materials	Particle Size (µm)	References
Ti	19.1	[73]
	<20	[74]
	74.9	[75]
	33.2	[153]
	75	[154]
	5	[155]
	<45	[156,157,158]
	45	[159]
	26.5	[160]
Hydride-dehydride (HDH) Ti	45	[46]
	29	[68]
	<77	[161]
	35.43	[162]
Ti-12Mo	HDH Ti: ≤45, hydrogen reduced Mo: ≤25	[163]
Ti-Mn	<45	[164,165]
Ti-Nb	Ti: <45, Nb: <110	[166]
	Ti: 21, Nb: <75	[167]
	Ti: 32.95, Nb: 30.15	[168]
	Ti: <14, Nb: <36	[169]
	Ti: 21, HDH Nb: 75	[170]
Ti-6Al-4V	13.4	[19]
	<45	[171]
	15	[172]
	25.5	[173]
	18	[174]
HDH Ti-6Al-4V	51.8	[47]
	70	[147]
Ti-6Al-4V/ Hydroxyapatite (HA)	Ti-6Al-4V: 19.54, HA: 61.95	[175]
	Ti-6Al-4V: 19.6, HA: 5	[176]
Ti-6Al-4V/Wollastonite (WA)	Ti-6Al-4V: 19.54, WA: 10.10	[177]
Ti-16Nb-(0-4) Sn	Ti: 23.81, Nb: 14.98, Sn: 20.19	[178]
Ti-24Nb-4Zr-8Sn	<45	[113]
Ti-27.5Nb-8.5Ta-3.5Mo-2.5Zr-5Sn	6.08	[55]

**Table 3:** Binders used in MIM processes pertaining to biomedical applications (years: 2013–2022).

Materials	Binders	References
Ti	Paraffin wax, polyethylene, stearic acid	[73]
	Polyethylene glycol 1500, poly methyl methacrylate, stearic acid	[75,154]
	Paraffin wax, high-density polyethylene, stearic acid	[153]
	Polyvinyl butyral, benzyl butyl phthalate, solsperse 20,000	[155]
	Paraffin wax, high-density polyethylene, stearic acid	[156]
	Polyethylene glycol, poly methyl methacrylate, polyvinylpyrrolidone	[157]
	Polyethylene glycol, polypropylene carbonate, poly methyl methacrylate	[158]
	Polyethylene glycol, poly methyl methacrylate, stearic acid	[159]
	Polyacetal-based thermoplastic binder	[160]
HDH Ti	Paraffin wax, high-density polyethylene, stearic acid	[46]
	Paraffin wax, low-density polyethylene, stearic acid	[68]
	Wax-based binders	[161]
	Agar, sucrose	[162]
Ti-12Mo	Liquid paraffin wax, stearic acid, low-density polyethylene, polypropylene, Polyethylene glycol, naphthalene, solid paraffin	[163]
Ti-Nb	Paraffin wax, polyethylene vinyl acetate, stearic acid	[167,170]
	Paraffin wax, carnauba wax, polypropylene, stearic acid	[168]
	Paraffin wax, low-density polyethylene, stearic acid	[169]
Ti-6Al-4V	Polyethylene glycol, polypropylene, stearic acid	[19]
	Paraffin wax, polyethylene vinyl acetate, stearic acid	[171]
	Polyoxymethylene, stearic acid, paraffin wax, ethylene vinyl acetate, polyethylene	[172]
	Paraffin wax, polypropylene, polyethylene, stearic acid	[173]
	Palm stearin, polyethylene	[174]
HDH Ti-6Al-4V	Polyethylene glycol, polyvinyl butyral, stearic acid	[47,147]
Ti-6Al-4V/HA	Palm stearin, low-density polyethylene	[175,195]
	Palm stearin, polyethylene	[176]
Ti-6Al-4V/WA	Palm stearin, polyethylene	[177]
Ti-16Nb-(0-4) Sn	Paraffin wax, carnauba wax, polypropylene, stearic acid	[178]
Ti-24Nb-4Zr-8Sn	Paraffin wax, polyethylene-vinyl acetate co-polymer, stearic acid	[113]
Ti-27.5Nb-8.5Ta-3.5Mo-2.5Zr-5Sn	Polyacetal-based binder	[55]

**Table 4:** Powder loading for titanium and titanium alloys (years: 2013–2022).

Materials	Particle Size (µm)	Powder Loading (vol.%)	References
Ti	19.1	72, 75, 80	[73]
	75	58	[154]
	<45	69	[156]
	<45	67	[157,158]
	45	60	[159]
	26.5	65.6	[160]
	28.4	67	[173]
HDH Ti	45	61	[46]
	29	53	[68]
	<77	55	[161]
Ti-12Mo	HDH Ti: ≤45, hydrogen reduced Mo: ≤25	65	[163]
Ti-Nb	Ti: 32.95, Nb: 30.15	50	[168]
	Ti: <14, Nb: <36	60	[169]
Ti-6Al-4V	13.4	60	[19]
	25.5	64	[173]
	18	65	[174]
HDH Ti-6Al-4V	51.8	55, 60	[47]
	70	55, 60	[147]
Ti-6Al-4V/HA	Ti-6Al-4V: 19.61, HA: 20	68, 69, 70	[209]
	Ti-6Al-4V: 19.54, HA: 61.95	68	[175]
	Ti-6Al-4V: 19.6, HA: 5	78.21	[176]
Ti-6Al-4V/WA	Ti-6Al-4V: 19.54, WA: 10.10	67	[177]
Ti-16Nb-(0-4) Sn	Ti: 23.81, Nb: 14.98, Sn: 20.19	50	[178]
Ti-24Nb-4Zr-8Sn	<45	65	[113]
Ti-27.5Nb-8.5Ta-3.5Mo-2.5Zr-5Sn	6.08	65	[55]

**Table 5:** Solvent debinding parameters utilized for titanium and titanium alloys (years: 2013–2022).

Materials	Solvent	Temperature (°C)	Time (h)	References
Ti	Hexane	50	24	[73,153]
	Water	50	35	[154]
	Hexane	50	20	[156]
	Water	40	28	[157]
	Water	50	–	[159]
HDH Ti	Hexane	50	20	[46]
	Heptane	45, 60, 75	6.5	[68]
Ti-12Mo	Heptane	50	15	[163]
Ti-Nb	Hexane	40	20	[166,167]
	Heptane	60	8	[168]
	Heptane	50	20	[169]
	Hexane	40	20	[170]
Ti-6Al-4V	Water	60	24	[19]
	–	40	15	[171]
	Heptane	60	6	[174]
Ti-6Al-4V/HA	Heptane	60	6	[175]
Ti-6Al-4V/WA	Heptane	60	6	[177]
Ti-16Nb-(0-4) Sn	Heptane	60	8	[178]
Ti-24Nb-4Zr-8Sn	Hexane	40	20	[113]

**Table 6:** Thermal debinding parameters for titanium and its alloys (years: 2013–2022).

Materials	Thermal Debinding Schedule	References
Ti	500 °C for 2 h	[73,153]
	450 °C for 1 h	[75,160]
	550 °C for 1 h	[156]
	570 °C for 2 h	[158]
HDH Ti	550 °C	[46]
	350 and 550 °C at 3 °C/min	[68]
Ti-12Mo	700 °C for 2 h	[163]
Ti-Nb	200–400–600 °C at 2 °C/min for 2 h	[168]
	250 °C for 2 h and 450 °C for 2 h	[169]
Ti-6Al-4V	700 °C at 2, 5, and 10 °C/min	[173]
	550 °C for 4 h	[174]
Ti-6Al-4V/ HA	500 °C	[175]
	320 °C at 3 °C/min for 1 h and 500 °C at 5 °C/min for 1 h	[176]
Ti-6Al-4V/WA	500 °C at 5 °C/min for 1 h	[177]
Ti-24Nb-4Zr-8Sn	500 °C	[113]
Ti-27.5Nb-8.5Ta-3.5Mo-2.5Zr-5Sn	500 °C for 2 h	[55]

**Table 7:** Sintering parameters used for titanium and its alloys (years: 2013–2022).

Materials	Sintering Temperature (°C)	Time (h)	Heating Rate (°C/min)	Sintering Atmosphere	References
Ti	1200	3	–	Vacuum	[73,153]
	1250	2	–	Argon	[75]
	1320	2	–	Argon	[154]
	1250	2	8	Argon	[155]
	1150, 1250, 1300	–	–	–	[156]
	1300	2	–	–	[157]
	1150	2	–	Vacuum	[160]
HDH Ti	1100–1300	2	–	Vacuum	[46]
	1350	2	6	Argon	[68]
	1150	2	–	Vacuum	[161]
Ti-Mn	1100	8	–	Vacuum	[164,165]
Ti-Nb	1500	4	–	Vacuum	[166]
	900–1500	2	5	Vacuum	[167]
	1500	4	10, 5	Vacuum	[168]
	1400	4	–	Argon	[169]
	1500	2	–	–	[170]
Ti-6Al-4V	1350	2	–	Vacuum	[171]
	1200, 1250, 1300	1, 2, 3	3, 4, 5	Vacuum	[174]
Ti-6Al-4V/HA	1350	3	–	Vacuum	[175]
	1100, 1200, 1300	2	–	Vacuum	[176]
Ti-6Al-4V/WA	1100, 1200, 1300	5	3	Vacuum	[177]
Ti-16Nb-(0-4) Sn	1250, 1400, 1550	2	10, 5	Vacuum	[178]
Ti-24Nb-4Zr-8Sn	1400, 1500	2, 4	–	Vacuum	[113]
Ti-27.5Nb-8.5Ta-3.5Mo-2.5Zr-5Sn	1000, 1100, 1200, 1300, 1400	8	–	Vacuum	[55]

**Table 8:** Mechanical properties of the sintered Ti and Ti alloy components (years: 2013–2022).

Materials	Sintering Temperature (°C)	Young’s Modulus (GPa)	Tensile Strength (Mpa)	Elongation (%)	References
Ti	1250, 1300	7.80, 22	–	–	[156]
	1300	–	617	–	[157]
	1150	99	542	–	[160]
HDH Ti	1250	–	395	12.5	[46]
Ti-8Mn	1100	87	–	–	[165]
Ti-9Mn	1100	89	1046	4.7	[165]
Ti-12Mn	1100	96	–	–	[165]
Ti-13Mn	1100	99	–	–	[165]
Ti-15Mn	1100	98	–	–	[165]
Ti-17Mn	1100	103	–	–	[165]
Ti-16Nb	1500	80	667	–	[167]
Ti-Nb	1500	100	–	–	[168]
Ti-17Nb	1400	76	–	–	[169]
Ti-6Al-4V	1350	–	824	–	[171]
	1200	–	934.33	–	[174]
Ti-6Al-4V + Gd	1350	–	749	–	[171]
Ti-6Al-4V/HA	1300	44.26	–	–	[176]
Ti6Al4V/WA	1100–1300	14.57–18.10	–	–	[177]
Ti-24Nb-4Zr-8Sn	1400	54	656	–	[113]
Ti-27.5Nb-8.5Ta-3.5Mo-2.5Zr-5Sn	1100	98	1154	–	[55]

## Data Availability

Not applicable.
